# Fluoride removal from aqueous solution via environmentally friendly adsorbent derived from seashell

**DOI:** 10.1038/s41598-022-13756-3

**Published:** 2022-06-10

**Authors:** Maryam Hashemkhani, Mohammad Rezvani Ghalhari, Parnia Bashardoust, Sara Sadat Hosseini, Alireza Mesdaghinia, Amir Hossein Mahvi

**Affiliations:** 1 Department of Electrical and Computer and Environment Engineering, West Tehran Branch-Islamic Azad University, Tehran, Iran; 2 Department of Environmental Health Engineering, School of Public Health, Tehran University of Medical Sciences, Tehran, Iran; 3 Center for Solid Waste Research, Institute for Environmental Research, Tehran University of Medical Sciences, Tehran, Iran

**Keywords:** Environmental sciences, Chemical engineering

## Abstract

Nowadays, the presence of excessive ions in water resources is of utmost concern and has attracted increasing attention; therefore, excessive amounts of these ions such as fluoride should be removed from drinking water. Conventional water treatment processes are shown to be incapable of the complete removal of redundant fluoride from aqueous water bodies, whereas adsorption is a promising, effective, cost–benefit, and simple method for this purpose. This study aimed to synthesize effective adsorbents from bivalve shells and evaluate the adsorption function of bivalve shells in removing fluoride from aqueous solutions. In this study, the oyster shell was collected from the Persian Gulf’s seaside and were crushed by manual mortar and blender, and graded with standard sieves with 70 mesh size. The prepared bivalve shell was characterized by SEM and FTIR. To investigate and optimize various variables on fluoride removal percentage a response surface methodology based on central composite design (RSM-CCD) was used. Under optimal conditions (pH: 5.5, adsorbent dose: 0.3 g/L, contact time: 85 min and fluoride concentration: 3 mg/L) the maximum removal efficiency was 97.26%. Results showed that the adsorption equilibrium and kinetic data were matched with the isotherm Langmuir Model (R^2^ = 0.98) with q_max_ = 27.31 mg/g and pseudo-second-order reaction (R^2^ = 0.99). Also, a thermodynamic study exhibited that the adsorption process of fluoride into bivalve shells was an exothermic reaction and could not be a spontaneous adsorption process. Based on the results, the bivalve shell was found as an appropriate adsorbent to remove fluoride from aqueous solutions.

## Introduction

Nowadays, concerns over the presence of excessive amounts of fluoride ions (F^−^), which is one of the earth's fundamental crust elements, in the drinking water bodies have spiked^1,2^. The World Health Organization (WHO) has recommended an appropriate range of F^−^ concentration for body intake between 0.5 and 1.5 mg/L. The stated guidelines can vary according to local and regional conditions (diet, bottled water usage, drinking water intake, etc.)^3,4^. As a result, in determining the appropriate fluoride daily intake, the area's climatic conditions, water volume, and other determining factors must be precisely specified^5^.

The high level of F^−^ concentration has many adverse effects on human health^6^, such as fluorosis^7^, abortion^8^, fertility disorders^9^, increased risk of bone, stomach, chronic kidney disease, thyroid-related problems^10–12^, damage to liver and kidney function in children^13–15^, decreasing children’s intelligence quotient^16^, the elevation of blood glucose, increased diabetes^17^, and gastrointestinal symptoms^18^. Also, a low F^−^ concentration (0.5 mg/L) has beneficial effects on teeth and bones. Since the body's fluoride is mainly supplied through food and toothpaste, the redundant fluoride concentration in drinking water supplies should be removed as much as possible to reduce the fluoride body intake from drinking water. Today, various methods can be applied for fluoride removal from aqueous solution, such as reverse osmosis and nanofiltration^19^, coagulation/chemical precipitation^20^, ion exchange^21^, and adsorption^22^, which each of these mentioned methods is selected according to water quality, economic parameters, and simple operation.

Conventional water treatment processes (e.g. coagulation, flocculation, and straightening) are shown to be incompetent for the complete removal of redundant fluoride from an aqueous solution, and many studies have shown that the adsorption method is a promising, effective, cost–benefit, and simple method for this purpose^23^. Recent studies have shown that due to the presence of a higher surface area of nanostructured materials and particles, and also greater amounts of active sites for interaction, the application of these nanomaterials as an absorbent could lead to better removal of pollutants from aqueous solutions^24^. However, there are several challenges related to the adsorption process such as higher cost of fabrication, difficulty in scaling up the synthesized nanoparticles, and selecting the suitable adsorbent according to the pollutant’s characterization. In recent decades using green adsorbents to remove various pollutants was increased, so based on their properties they are efficient, recyclable, and environmentally friendly, which for these beneficial factors can be used widely^25–27^. Recently, using calcium components (i.e. calcium phosphate) as an adsorbent in fluoride removal has been extensively discussed^28^.

Good features such as high availability, ionic exchange property, adsorption affinity, and its capability to establish bonds with various organics of different sizes make calcium-derived adsorbents a good candidate for the adsorption processes^29^. Furthermore, these adsorbents can be used in batch or continuous column systems^30^. Since the remains of the seashells contain high amounts of various elements including silica and calcium, it can be considered one of the main sources for the synthesis of calcium adsorbent for the defluorination process due to its natural origin, easy access, and economic property. Bivalves, one of the remains, are the second-largest mollusks with bilateral symmetry and can be found in saline and sweet waters. Around 95% of the structure of Bivalves consists of calcium^31,32^. The bivalve’s skeletal shells can be found in huge amounts (tons) on the coastline of southern cities of Iran. These materials are mostly thrown away and a lot of money is needed for the proper disposal of these materials. As a highly available material, the bivalve shells are a good candidate for synthesizing a cost-effective adsorbent, and using them will also reduce the expenses related to proper disposal of them; therefore, the aims of this study are to (1) synthesize effective adsorbents from bivalve shells and (2) evaluate the adsorption function of bivalve shells in removing fluoride from aqueous solutions.

## Material and methods

The present study is an experimental laboratory scale conducted in a batch flow system. This study has investigated the application of Bivalve Shell adsorbent for the defluorination process from aqueous solutions by the change in independent variables such as pH, contact time, adsorbent dosage, fluoride concentration, and temperature.

### Materials and reagents

Unless otherwise stated, all chemicals used in this study, such as sodium hydroxide (NaOH, 1 M), Hydrochloric acid (HCl, 1 M), Tetrachloride Ferric (FeCl_2_·4H_2_O), Hexa Chloride Ferric (FeCl_3_·6H_2_O), sodium fluoride (NaF) zirconyl oxychloride, and sodium 2-(para-sulfophenyl azo)-1, 8-dihydroxy-3, 6-naphthalene disulfonate (SPADNS) were in the analytical reagent grade (AR) and were purchased from Merck Company located in Germany. All chemicals used in this study were used without further purification. The devices used in this study were: Hack Spectrophotometer (DR/2000, made in the USA), Innova 4340 Incubator, GFL3018 Shaker, and Kent EIL7020 pH-meter (made in German). In this study, HCl (1 N) and NaOH (0.1 N) were used for pH adjustment, and double deionized water (Milli-Q Millipore 18.2 MΩ cm^−1^ conductivity) was used for all solutions. The stock solution was prepared by dissolving appropriate amounts of sodium fluoride in deionized water. Further dilution was then done to obtain the required initial concentration solutions. 1 M NaOH or 1 M HCL solutions were used for the adjustments of pH.

### Adsorbent preparation and characterization

The oyster shell, which is one of the most common two-species oysters available at the Persian Gulf’s seaside, was used in this study. The collected mussel shells were rinsed several times with distilled water and placed in the oven at 105 °C for 48 h to remove moisture. Dried bivalve shells were crushed by manual mortar and blender and graded with standard sieves with 70 mesh size.

Fourier transform infrared (FT-IR, Nexus TM 670) was used to determine the chemical structure of the bivalve shell in 4000–400 cm^−1^. To determine the surface and morphology characteristics of the bivalve shell, scanning electron microscopy (SEM; Hitachi S-4700, Tokyo, Japan) was used.

### Batch adsorption and experimental design

The independent variables (pH, adsorbent dosage, contact time, and initial fluoride concentration) affect the dependent variable (efficiency of bivalve shell on fluoride removal) and the optimum conditions were investigated using the R software (*version R i 386 4.1.2*) by applying response surface methodology (RSM) package. All adsorption experiments in this study were carried out with pH (3–9), initial concentration (2–12 mg/L), Time (10–90 min), and adsorbent dosage (0.1–0.5 g/L) on fluoride removal. To investigate the effects of the variables on the removal efficiency, the central composite design (CCD) was used (see Table 1). All experiments were carried out in a shaker (CFL 3018) and were performed at 150 rpm. To prepare a stock solution of fluoride (1000 mg/L), 0.0221 mg NaF was dissolved in 100 mL of double-distilled water. Based on Table 1, different concentrations of stock solutions were prepared. In all statistical analyses, *P* value < 0.05 was considered a significant value.

**Table 1:** CCD matrix ranges and their response to fluoride adsorption by bivalve shell.

Run order	pH	Adsorbent (g L^−1^)	Time (min)	Concentration (mg L^−1^)	Removal (%)
1	7.5	0.4	70	4.5	55.0
2	4.5	0.4	70	9.5	44.2
3	6	0.3	50	7	51.3
4	7.5	0.2	70	9.5	39.1
5	7.5	0.4	30	9.5	29.4
6	4.5	0.2	30	4.5	35.2
7	6	0.3	50	7	79.3
8	6	0.3	50	7	49.9
9	4.5	0.2	70	4.5	54.8
10	4.5	0.4	30	4.5	47.2
11	7.5	0.2	30	4.5	14.6
12	4.5	0.2	70	9.5	42.0
13	4.5	0.2	30	9.5	27.5
14	6	0.3	50	7	63.0
15	6	0.3	50	7	55.0
16	7.5	0.4	70	9.5	50.2
17	4.5	0.4	70	4.5	92.8
18	7.5	0.2	30	9.5	7.2
19	7.5	0.2	70	4.5	50.0
20	4.5	0.4	30	9.5	54.0
21	6	0.3	50	7	54.3
22	6	0.3	50	7	52.3
23	7.5	0.4	30	4.5	57.9
24	6	0.3	50	7	50.6
25	9	0.3	50	7	3.3
26	6	0.3	50	7	73.0
27	6	0.3	50	7	55.4
28	6	0.3	50	7	56.9
29	6	0.3	50	2	87.0
30	6	0.3	50	7	53.0
31	6	0.3	10	7	19.4
32	6	0.1	50	7	24.9
33	6	0.3	90	7	98.4
34	6	0.3	50	7	78.1
35	6	0.5	50	7	83.9
36	3	0.3	50	7	54.6
37	6	0.3	50	7	76.4
38	6	0.3	50	7	69.9
39	6	0.3	50	12	49.8

SPADNS reagent method was used for the measurement and analysis of fluoride. To prepare the SPADNS solution, 958 mg of SPADNS was dissolved in 500 mL of doubled distilled water. Then, the zirconium acid reagent was prepared by adding 133 mg of zirconium chloride to 25 mL of doubled distilled water, then 350 mL of HCl 1 N was added and diluted to 500 mL with distilled water. To prepare the reference solution, 10 mL of the SPADNS solution was added to 100 mL of doubled distilled water. Then, the diluted HCl was added to the SPADNS solution. Finally, SPADNS solution and zirconium acid reagent were mixed in equal proportions.

The pH was adjusted using sodium hydroxide (NaOH) 0.1 N and hydrochloric acids (HCl) 0.1 N solutions and all samples were checked via a portable pH meter (Kent EIL 7020). 50 mL of the fluoride solution was used to perform batch equilibrium adsorption experiments. After the adsorption process, in order to separate the adsorbent from samples centrifugation method (5000 rpm and 15 min) (Sigma 2-16KL) was used.

In this study, Fluoride ion measurement was performed using the standard SPADNS method and spectrophotometer (DR 5000 Company, U.S.A.) at 570 nm wavelength. After each adsorption experiment, the fluoride removal percentage and adsorption capacity were calculated by using Eqs. (1) and (2).

1 $$\%Adsorption= \frac{\left({C}_{0}-{C}_{e}\right)}{{C}_{0}}\times 100$$

2 $$Qe= \frac{({C}_{0}- {C}_{e})\times V}{M}$$

where C_0_ is the initial fluoride concentration, C_e_ is residual fluoride concentration for each run by consideration designed experiment, Q_e_ is the amount of fluoride absorbed per unit mass of adsorbent (mg g^−1^), and V demonstrated the volume of fluoride solution (L), and M is an adsorbent dose which used in each run (g).

### Adsorption isotherms, kinetic study, and thermodynamic

In the current study, to evaluate the interaction between fluoride molecules and bivalve shell Langmuir, Freundlich, Temkin, and Dubinin–Radushkevich (D–R) isotherm models were investigated which are described in Table 3. In the isotherm model investigation, all parameters were adjusted at equilibrium conditions except for the initial concentration of the fluoride which varied. Determination of the applied adsorption isotherms and adsorption capacities are the most important parameters for estimating system performance. Linear equations of states were used to check the fit of the data to the absorption isotherm models.

The reaction order of fluoride adsorption processes was described by Kinetic models including first-order equation^33^ second-order expression^34^, Elovich (E)^35^, and intraparticle diffusion (ID)^36^ models described in Table 3. Also, The adsorption process of fluoride on the bivalve shell was investigated at different temperatures, including 283, 293, 303, and 313 °K and the thermodynamic model was calculated based on Eqs. (3) to (5)^37^:

3 $${\Delta G}^{\circ }=-RTLn{K}^{\circ }$$

4 $$\mathrm{ln}\left(\frac{qe}{Ce}\right)=\frac{{\Delta S}^{\circ }}{R}-\frac{{\Delta H}^{\circ }}{RT}$$

5 $${\Delta G}^{\circ }={\Delta H}^{\circ }-T{\Delta S}^{\circ }$$

By which the Gibbs free energy was described by ∆G°, the gas constant (8.314 J mol^−1^ K^−1^) was defined by R, the temperature considered as kelvin degrees and was defined by T, K° was the thermodynamic equilibrium constant, C_e_ was the residual concentration of the fluoride in the solution (mg L^−1^), the enthalpy changes were described by ΔH° and the entropy changes were defined by ΔS°.

### Ethical approval

Not applicable.

### Consent to participate

Not applicable.

### Consent to publish

Not applicable.

## Results and discussion

### Characterization of the bivalve shell

**Figure 1:**
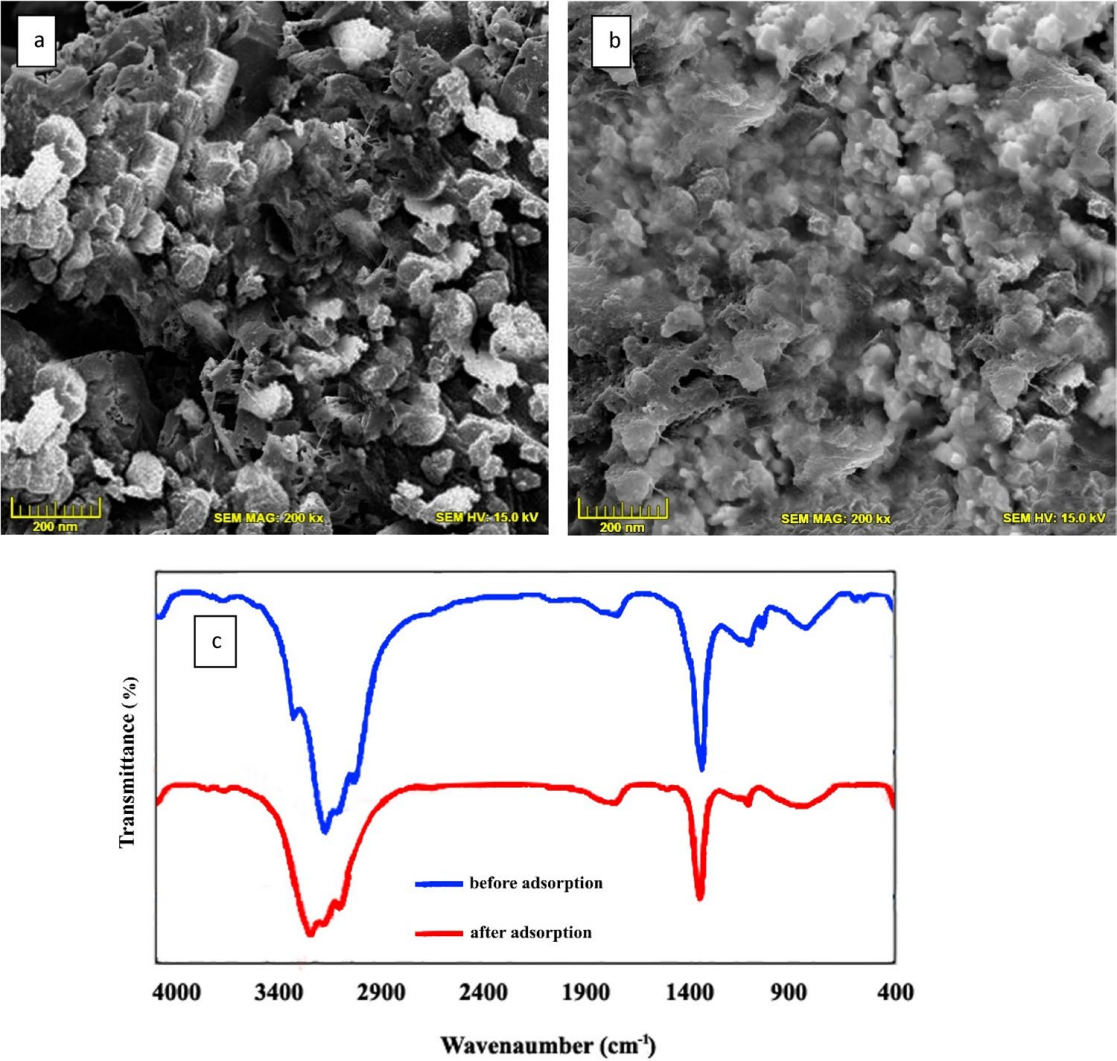
SEM images of bivalve shell before (**a**) and after adsorption (**b**) and FT-IR analysis (**c**) of adsorbents.

In Fig. 1 SEM images of the bivalve shell are shown. Figure 1a shows the bivalve shell before fluoride adsorption in which the black and white dots represent the adsorbent porous which are appropriate sites for pollutant molecules. As shown in Fig. 1b these sites were occupied by fluoride molecules after the fluoride adsorption process was performed; also, it can be seen that the bivalve shell has multi-dimensional structures, the surface of the adsorbent was jagged, the bivalve shell’s structure did not have a uniform shape, and a little agglomeration was observed in it. The mentioned properties can enhance the contact between the fluoride ions and the synthesized adsorbent, eventually leading to better adsorption performance. Also, the FT-IR spectrum of bivalve shells before and after the adsorption process shows that there are two strong absorbance peaks, all the FTIR spectra looks the same but the distribution and minimum peaks were different, one of them was around 3351 cm^−1^ representing the characteristic stretching of O–H stretching, and the other peak was around 1351 cm^−1^ which is characteristic. C–H bending and C–H stretching in CH_3_ may correspond to Ca(OH)_2_ formation^38^.

### CCD modelling

In this study, the RSM-CCD approach and ANOVA test were used to validate the model and operating parameters. Table 2 reports the results obtained from a statistical analysis of the operating parameters. A low *p* value (< 0.001) and an appropriate value of lack of fit (0.26) showed that the developed model was significant. Also, coefficient of determination values of, i.e., R^2^ > 0.74 and R^2^_adj._ > 0.69. the difference between R^2^ and R^2^_adj._ was less than 0.2, demonstrating a high degree of accuracy and reliability of the selected model^39^.

**Table 2:** ANOVA test for CCD modeling and results of process optimization.

	Estimate	Std. Error	t value	Pr( >|t|)	Sig
**Parameters statistics**					
(Intercept)	59.5917	2.6471	22.5122	**< 0.001**	***
pH	− 16.4083	4.9522	− 3.3133	**0.0022**	**
Adsorbent dose	23.1917	4.9522	4.6831	**< 0.001**	***
Contact time	26.0917	4.9522	5.2687	**< 0.001**	***
Concentration	− 15.6917	4.9522	− 3.1686	**0.0033**	**
pH*pH	− 38.5813	8.423	− 4.5805	**< 0.001**	***
Time*Time	− 8.6312	8.423	− 1.0247	0.3131	

	Df	Sum Sq	Mean Sq	F value	Pr(> F)
**Model statistics**					
FO (pH, adsorbent dose, contact time, concentration)	4	10,404.5	2601.14	17.6769	< 0.001
PQ (pH, contact time)	2	3297.5	1648.74	11.2046	< 0.001
Residuals	32	4708.8	147.15		
Lack of fit	18	3023.6	167.98	1.3956	**0.2663**
Pure error	14	1685.1	120.36		

		Value		Unit	
**Optimization process**					
pH		**5.5**			
Adsorbent		**0.3**		g L^−1^	
Time		**85**		min	
Concentration		**3**		mg L^−1^	
Predicted efficiency by the solver		**97.26**		%	

Significant values are in bold.

To predict and optimize response for the given levels of each independent factor on efficacy regression equation in terms of actual factors was used. The quadratic equation corresponding to the CCD model for fluoride adsorption on bivalve shells can be defined as shown below:

$$\begin{aligned} {\text{Y}} & = 59.5917 - 16.4083\;\left( {{\text{pH}}} \right) + 23.1917\;\left( {\text{Adsorbent dose}} \right) + 26.0917\;\left( {\text{Contact time}} \right) \\ & \quad - 15.6917\;\left( {{\text{Concentration}}} \right) - 38.5813\;\left( {{\text{pH}}*{\text{pH}}} \right) - 8.6312\;\left( {{\text{Time}}*{\text{Time}}} \right) \\ \end{aligned}$$

### Effect of pH on fluoride adsorption

**Figure 2:**
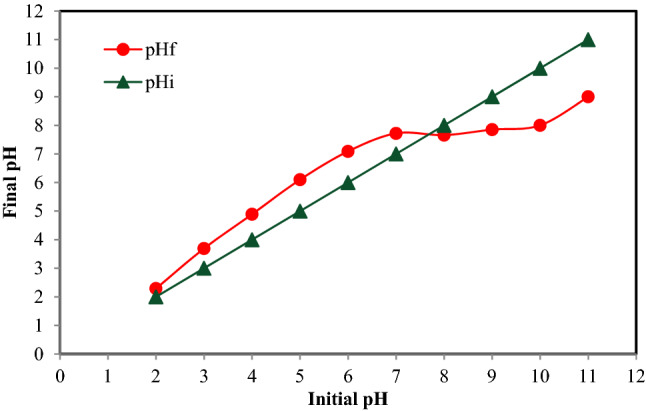
pH_pzc_ of the synthesized adsorbent.

**Figure 3:**
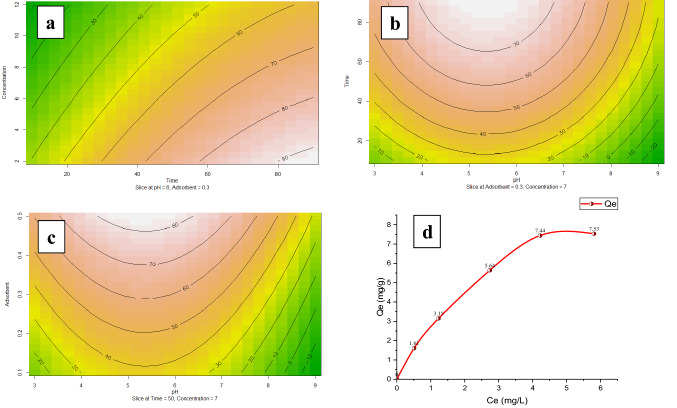
Contour plots and main effect of variables [Contact time and Fluoride concentration (**a**), Contact time and pH (**b**), pH and Adsorbent dose (**c**)] on fluoride removal (%) and (**d**) adsorption capacity of the bivalve shell.

pH is one of the most critical factors affecting the process efficiency and ionization of the material. pH can affect the adsorbent surface charge and acid or base ionization which eventually can affect the adsorption action. Many studies have shown that the maximum adsorption occurs at the isoelectric point (when the compound has zero charges)^40,41^. In acidic conditions, the hydrogen ions move to the adsorbed molecules, creating a positive area on the surface and reducing the uptake of positive ions as a competitor. The pH_pzc_ of the synthesized adsorbent was 7.6; the results of pH_pzc_ is depicted in Fig. 2. In acidic conditions, the presence of a positive charges on the surface of the synthesized adsorbent will favor the electrostatic attraction of the fluorides. As shown in Fig. 3, fluoride adsorption was increased by increasing the pH value, and The best adsorption capacity for molecules occurred at pH 5.5. Whereas after pH 5.5, the adsorption capacity was reduced because added OH ions can change fluoride molecules to fluoride ions. Since the synthesized adsorbent is mainly consisted of CaCO_3_, when placed in acidic conditions, they will turn into Ca^2+^ and CO_3_^2−^, and the obtained ions will favor the precipitation reaction of fluorides^42^. The results showed that at pH 5.5, the best adsorption of fluoride into bivalve shells happens, which was compatible with Samadi et al.^43^ who used active alumina, Campbell et al.^44^, who used modified chitosan and Asgari et al.^45^ who used cochlear shell to adsorption of fluoride.

### Effect of adsorbent dosage on fluoride adsorption

The adsorbent dose is another parameter that was investigated in this study. Increasing the adsorbent dosage can increase the fluoride removal rate since the available sites for the adsorption of pollutants are increased. As shown in Fig. 3 optimum value of the adsorbent was 0.3 mg/g. Under optimum conditions, fluoride adsorption can be increased to 97.6%, which was compatible with Parastar et al.^46^ and Zazouli et al.^47^.

### Impact of contact time on fluoride adsorption

Figure 3 shows that with the increase in contact time, the removal efficiency increased and reached equilibrium after 85 min. Fluoride removal was increased because there is enough time for the fluoride molecules to connect with the adsorbent sites. Also, the fluoride removal curve has a soft slope over time, which may be due to the formation of a thin layer of fluoride on the adsorbent surface. The contact time in the present study was more than the Adak et al.^48^ study which used Al(III)–Fe(III)–La(III) trimetallic oxide as an adsorbent for fluoride, but the bivalve shells are natural adsorbents that have limited sites for the removal of pollutants. Whereas Adak et al.^48^ used a synthetic adsorbent derived from chemical substances that can make multitudinous porous among adsorbents. The results of this study were in accordance with Zarrabi et al.^49^ who showed that the adsorption process and contact time have a positive correlation.

### Effect of initial fluoride concentration on adsorption

The effect of the initial concentration of fluoride on the rate of fluoride removal by the bivalve shell was investigated. As shown in Fig. 3, the most fluoride adsorption occurred in the lowest initial concentration (2 mg L^−1^) in which, the fluoride adsorption efficiency was more than 90%. The adsorption efficacy was reduced by increasing the initial concentration because fluoride molecules occupied the adsorbent sites. To obtain more adsorption efficacy, more adsorbent dosage and more contact time were needed. The results were matched with Zazouli et al.^47^, and Tor et al.^50^ showed that by increasing the fluoride's initial concentration, the fluoride removal efficiency decreased.

### Equilibrium capacity

As shown in Fig. 3d the equilibrium adsorption isotherm of fluoride by bivalve shell were investigated by considering optimum experimental conditions (pH = 5.5; Dosage = 0.3 g L^−1^; and contact time = 85 min). First, the fluoride adsorption rate into the bivalve shell was increased linearly (first-order) with the initial fluoride concentration. By increasing the fluoride concentration at the initial concentration of 4.5–6 mg L^−1^, the curve changes to a flat curve and reaches maximum adsorption capacity (7.53 mg g^−1^), which is indicant the actual adsorption capacity.

### Adsorption isotherms

**Figure 4:**
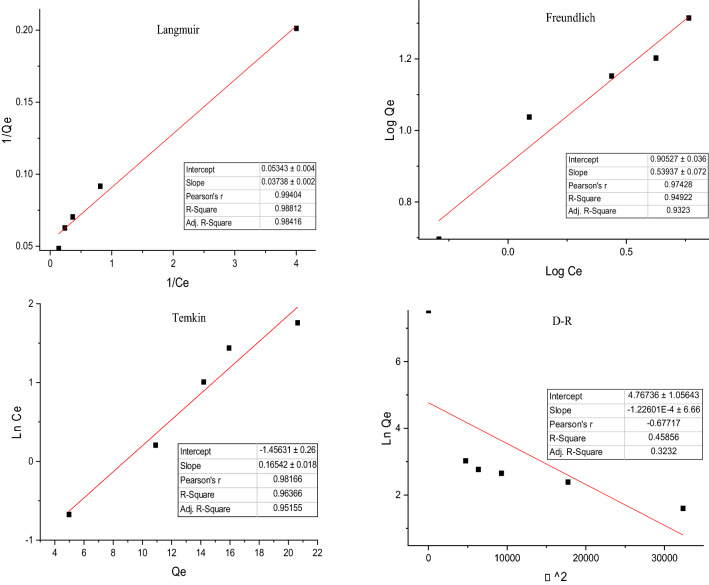
Linear (**a**–**d**) curves of the isotherm models of fluoride adsorption on the bivalve shell under the optimized condition.

**Figure 5:**
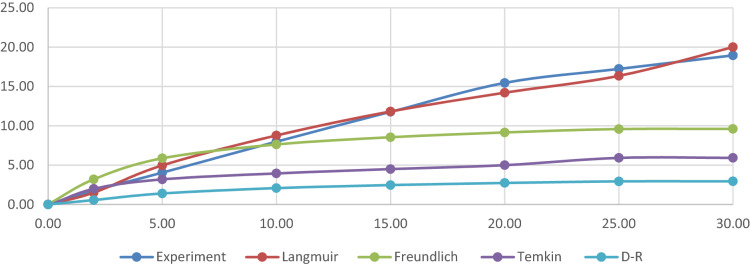
Linear curves and non-linear of the isotherm models of fluoride adsorption on the bivalve shell under the optimized condition.

In this section, in order to evaluate the interaction between the bivalve shell’s porous and fluoride molecules, and also the adsorption mechanisms, the mentioned isotherm models in “Adsorption isotherms, kinetic study, and thermodynamic” section. by considering Table 3 content, were performed. Figures 4 and 5 and Table 3 show that by considering the correlation coefficient, the experimental data of fluoride adsorption into bivalve shell fit well with the Langmuir model (R^2^ = 0.9881 and Pearson’s r = 0.994); from these results, it can be deducted that the interaction between fluoride molecules in the prepared solution was significantly weak and also the adsorption mechanisms of fluoride molecules into bivalve shell was as a monolayer. The results of the isotherm models in the present study were matched by Meliani et al.^51^, Lee et al.^52^, Lee et al.^53^, Asgari et al.^45^, and Parastar et al.^46^, which the fitted isotherm model was more suitable with Langmuir isotherm.

**Table 3:** Isotherm, kinetic, and thermodynamic parameters for fluoride adsorption into bivalve shell under the optimized condition.

Model	Equation	Parameters		Fluoride values	
**Isotherms**					
Langmuier	C_e_/q_e_ = C_e_/Q_m_ + 1/K_a_Q_m_	Q_m_ (Fluoride g^−1^)		27.31	
		K_L_ (L mg^−1^)		0.45	
		Slope		0.03738 ± 0.002	
		Intercept		0.05343 ± 0.004	
		R^2^		**0.98812**	
		Pearson’s R		0.99404	
Freundlich	ln q_e_ = (1/n)ln C_e_ + lnK_F_	n		1.85	
		K_F_ (L mg^−1^)		8.04	
		Slope		0.53937 ± 0.072	
		Intercept		0.90527 ± 0.036	
		R^2^		0.94922	
Temkin	q_e_ = B_l_ ln C_e_ + B_l_ ln K_T_	B_l_		0.1654	
		K_T_ (L mg^−1^)		0.000150188	
		Slope		0.16542 ± 0.018	
		Intercept		− 1.45631 ± 0.26	
		R^2^		0.96366	
Dubinin and Radushkevich	ln q_e_ = − Kε^2^ + ln Q_s_	β		− 4.7418E−05	
		Qm		3.15583795	
	(ε = RT ln (1 + 1/C_e_))	Slope		− 1.22E−04 ± 6.66	
		Intercept		4.767 ± 1.05643	
		R^2^		0.45856	
**Kinetics**					
First-order kinetic	ln(q_e_ − q_t_) = − k_1_t + ln(q_e_)	k_1_(min^−1^)		0.07	
		q_e_ (Fluoride/g)		17.61	
		Slope		− 0.07492 ± 0.009	
		Intercept		2.86845 ± 0.53	
		R^2^		0.94452	
Second-order kinetic	t/q_t_ = t/q_e_ + 1/(k_2_q_e_)^2^	k_2_(g/mg^-1^ min^−1^)		0.007475	
		q_e_ (Fluoride/g)		10.44	
		Slope		0.09575	
		Intercept		1.2265 ± 0.183	
		R^2^		**0.99592**	
Intraparticle diffusion	q_t_ = K_diff_ t^1/2^ + C	K_i_		− 2.49	
		K_p_		0.59	
		Slope		0.59062 ± 0.1	
		Intercept		3.74771 ± 1	
		R^2^		0.80415	
Elovich	q_t_ = 1/β ln(t) + 1/β ln(αβ)	β (g mg^−1^)		2.07	
		α (g mg^−1^ min^−1^)		0.25	
		Slope		2.06825 ± 0.3	
		Intercept		0.03211 ± 1.21	
		R^2^		0.90205	

Temperature ◦K	ln kd	∆S° (kJ/mol K)	∆G° (kJ/mol)	∆H° (kJ/mol)	qe
**Thermodynamic**					
283	3.98	− 0.0399	− 9.365	− 296.505	5.89
293	3.53		− 8.611		5.83
303	3.22		− 8.117		5.77
313	3.16		− 8.236		5.757

Significant values are in bold.

### Kinetic study

**Figure 6:**
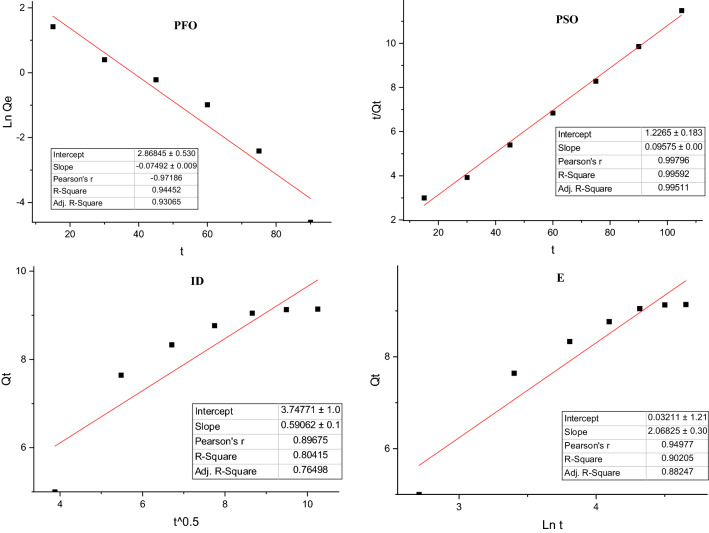
Linear (**a**–**d**) curves of the kinetic models of fluoride adsorption on the bivalve shell under the optimized condition.

In the present study, in order to investigate the adsorption behaviors of fluoride into bivalve shells at pre-selected time intervals, the mentioned kinetic models in “Adsorption isotherms, kinetic study, and thermodynamic” section, also described in Table 3, were performed. As shown in Fig. 6, the kinetic models of fluoride adsorption into bivalve shells by considering the linear regression test were fitted to the PSO kinetic model (R^2^ = 0.9959). PSO kinetic model predicts the behavior over the whole range of time studied; the fitted kinetic model with the PSO model means that the fluoride adsorption rate constant into bivalve shells depends on the initial fluoride concentration in the aqueous solutions. Hence, it means that there is a negative correlation between concentration and adsorption efficacy, and decreasing the initial fluoride concentration can help to increase the fluoride adsorption rate; these results were matched with Iriel et al.^54^ and Raghav et al.^55^ because of the adsorption process followed a PSO kinetic model. Kinetic parameters for all models are given in Table 3.

### Thermodynamic

**Figure 7:**
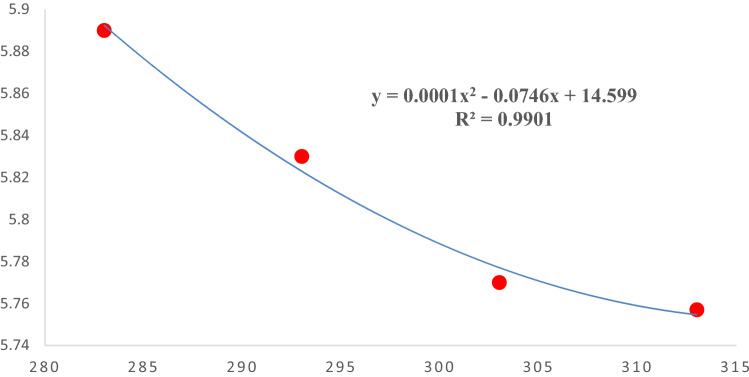
Effect of temperature on adsorption capacity of Bivalve Shell in Fluoride adsorption.

The temperature change can also affect the adsorption efficacy, so in the present study, a thermodynamic study was conducted to determine the effect of temperature on the fluoride adsorption efficacy and bivalve shell adsorption capacity by considering 283, 293, 303, and 313 °K, and using Eqs. which mentioned in “Adsorption isotherms, kinetic study, and thermodynamic” section. Figure 7 shows that by increasing the solution temperature, the adsorption efficacy was decreased (R^2^ = 0.9901), so there is a reverse linear association between temperature and adsorption efficacy. Also, Table 3 demonstrated that the value of the ΔH° was negative (ΔH° = − 296.505 kJ mol^−1^) means that fluoride's adsorption mechanism into bivalve shell was an exothermic reaction and followed the physical adsorption process and was matched with Lin et al. (2015). Table 3 shows that the adsorption process Gibbs Free energy (ΔG°) has a negative value in all situations and increases with increasing reaction temperature these negative values mean that the fluoride adsorption into bivalve shell can be a spontaneous adsorption process.

### Regeneration study

**Figure 8:**
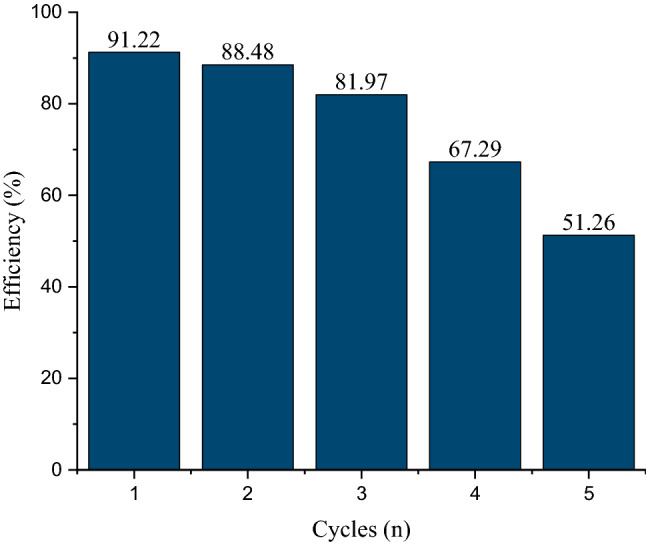
Reusability of the Bivalve Shell for Fluoride removal.

The regeneration research was performed by considering optimum condition (pH = 5.5, adsorbent dose = 0.3 mg g^−1^, contact time = 85 min and fluoride concentration = 3 mg L^−1^) and for desorption process the HCl 0.1 mol L^−1^ solution for 45 min was used to separate fluoride molecules from bivalve shell sites. the results of the regeneration study are shown in Fig. 8. This can be contributed to the fact that by increasing the number of cycles most of the adsorption active sites are filled. As a result, empty adsorption sites are out of reach, contributing directly to a decrease in removal efficiency.

## Conclusion

The present study demonstrated that the bivalve shell can be considered an effective adsorbent for the removal of excess fluoride from aquatic sources. This process was proven to be very efficient under optimal conditions (pH: 5.5, adsorbent dose: 0.3 g/L, contact time: 85 min, and fluoride concentration: 3 mg/L) in which the maximum removal of fluoride (97.26%) was achieved. The results indicated that the experimental data fitted well with the isotherm Langmuir Model. The kinetics of adsorption followed a pseudo-second-order model; and, the thermodynamic studies exhibited evidence exothermic nature of the adsorption process, which cannot be spontaneous. The overall results indicated that the bivalve shell is an effective method for the removal of fluoride from water sources.

## Data Availability

All data generated or analyzed during this study are included in this published article.
